# Case report: Congenital multidrug-resistant tuberculosis

**DOI:** 10.3389/ftubr.2024.1343217

**Published:** 2024-04-09

**Authors:** Hongyi Zhang, Lu Xia, Peize Zhang

**Affiliations:** Department of Pulmonary Disease, The Third People's Hospital of Shen Zhen, Shenzhen, Guangdong, China

**Keywords:** neonate, congenital tuberculosis, multidrug-resistant tuberculosis, diagnosis and treatment of tuberculosis, *in vitro* fertilization-embryo transfer (IVF-ET)

## Abstract

Congenital multidrug-resistant tuberculosis (MDR-TB) is a rare disease with high mortality. We report a case of a neonate girl with congenital MDR-TB. The infant's mother underwent *in vitro* fertilization–embryo transfer and did not show any symptoms prior to delivery. After the 14th day of life, the infant had a fever and worsening difficulty breathing despite antibiotic treatment. She was then confirmed to have congenital MDR-TB and received ventilation and anti-TB treatment. When the infant's TB was diagnosed, her mother was screened for TB and found to have MDR-TB, affecting both her lungs and reproductive system. They both recovered and were discharged from the hospital, with anti-TB treatment ongoing.

## Introduction

Congenital tuberculosis (TB) is tuberculosis caused by the infection of a fetus before or during delivery. It is a rare disease with high mortality rates. There are two ways of infection by *Mycobacterium tuberculosis*. During pregnancy, the bacteria can be transmitted vertically from the mother through the placenta or the umbilical cord, causing primary liver syndrome. The bacteria can spread hematogenously to the whole body of the fetus in this way; this type accounts for the majority of the cases (1). Another type is primary infection in the lungs or intestines, caused by the fetus inhaling or swallowing amniotic fluid, secretions, or blood containing *Mycobacterium tuberculosis* during delivery, resulting in the primary pulmonary infections, and then spreading through the bloodstream or lymph nodes throughout the body (2). The incidence rate of congenital tuberculosis in countries with high tuberculosis rates is approximately 2% (3). Congenital multidrug-resistant TB (MDR-TB) is defined by the World Health Organization as the *Mycobacterium tuberculosis* strain that is resistant to both isoniazid and rifampicin, which are the most effective first-line anti-TB drugs. The treatment outcome of MDR-TB remains suboptimal, despite the development of new drugs such as bedaquiline, linezolid, and delamanid in the last decade (4). We hereby report the diagnosis and treatment of a baby girl with congenital MDR-TB. She was confirmed to have congenital MDR-TB, and her mother was then found to have MDR-TB affecting both her lungs and reproductive system.

## Case presentation

A 22-day-old baby girl was hospitalized with a fever for 8 days. On the 14th day after birth, fever began with a maximum temperature of 38.5°C. The blood examination showed an increase in white blood cells and C-reactive protein, and the chest X-ray showed bilateral pneumonia lesions. She received empirical treatment with piperacillin, sulbactam, and meropenem, as well as oxygen therapy. However, the fever persisted, the inflammatory index increased, and chest X-rays showed a progression of the lesion. On day of life (DOL) 20, sputum tested positive for *Mycobacterium tuberculosis* DNA, with rifampicin and isoniazid (resistant). On DOL22, the baby was transferred to our hospital for further treatment. Past history records that the baby was born at 36 weeks 6 days. She weighed 2.43 kg at birth and had not received the Bacillus Calmette-Guérin (BCG) vaccination.

The admission examination data at our hospital were the following: T: 37.6°C, P: 165 times/minute, R: 27 times/minute, BP: 70/38 mmHg, SpO_2_: 82%, weight: 2.47 kg, normal development. There was no yellow staining of the skin and mucous membranes, and the lymph nodes were not palpable and swollen. The breathing sounds in both lungs were thick, with some moist rales. The abdomen was flat and soft, the liver was not palpable, and the bowel sounds were normal. The strength and tension in the muscles of the limbs were normal.

**Figure 1:**
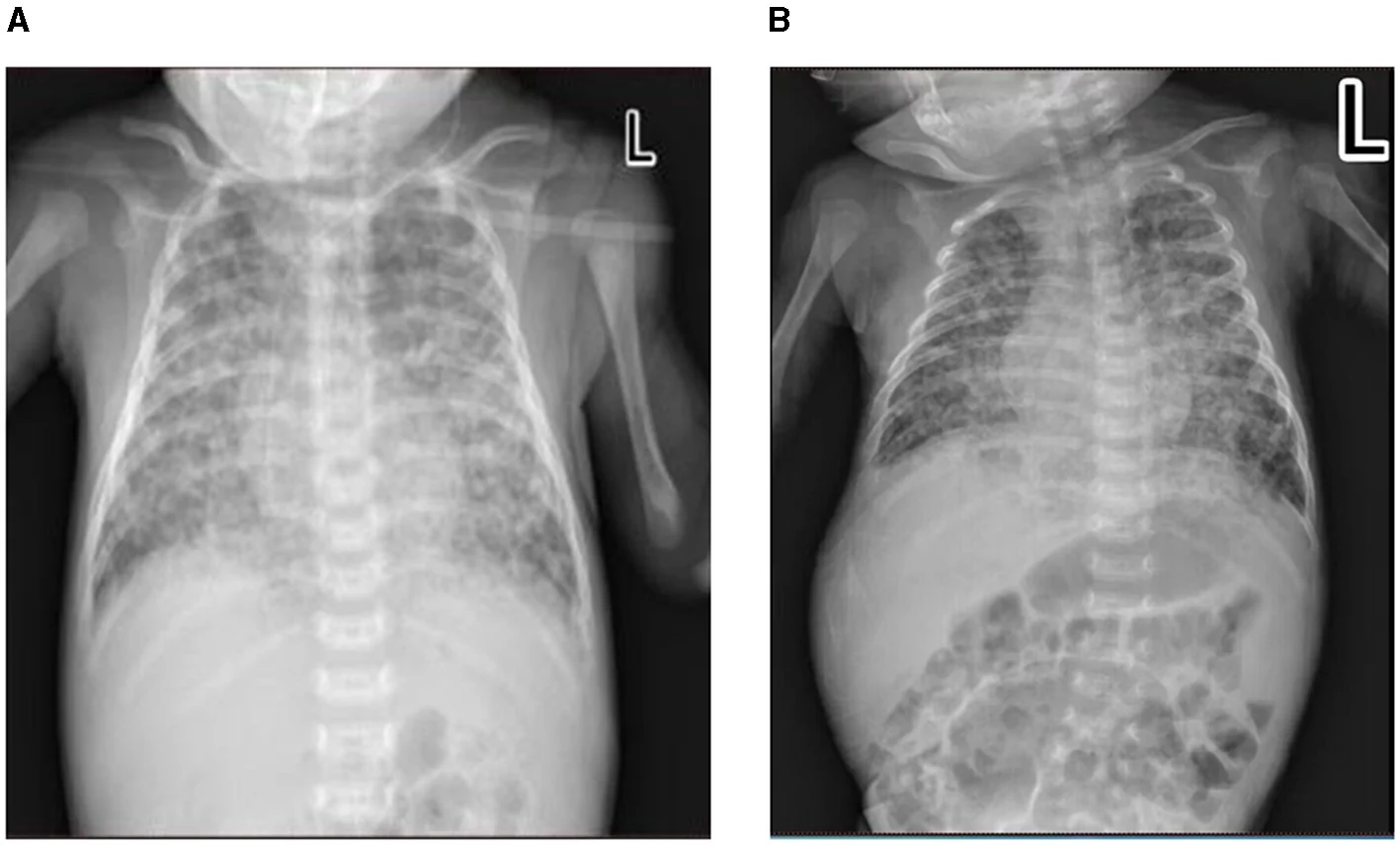
Chest X-ray of the patient at diagnosis of congenital tuberculosis (TB). **(A)** In the early stages of anti-TB treatment/day of life 25. **(B)** On the 26th day of anti-TB on day of life 47.

**Figure 2:**
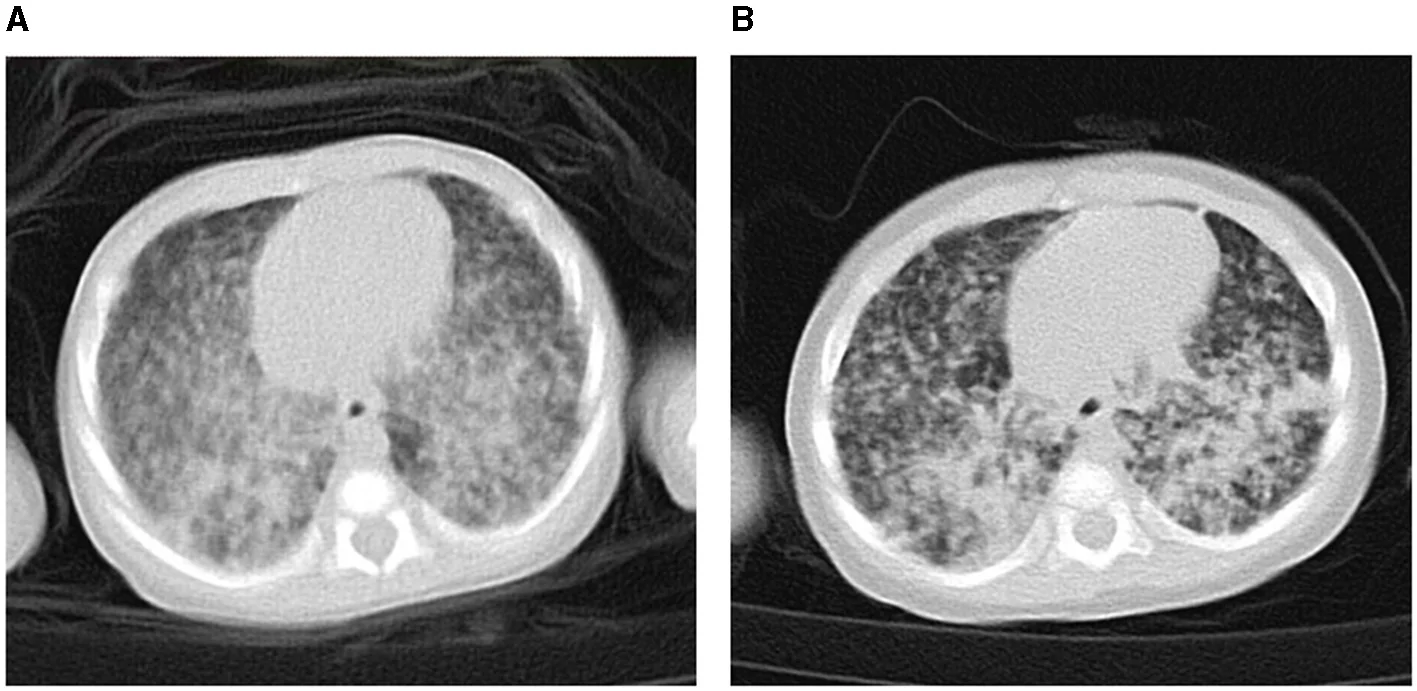
Chest computed tomography of the patient at diagnosis of congenital tuberculosis (TB). **(A)** On the 20th day of anti-TB treatment/day of life 41. **(B)** On the 70th day of anti-TB treatment/day of life 91.

**Figure 3:**
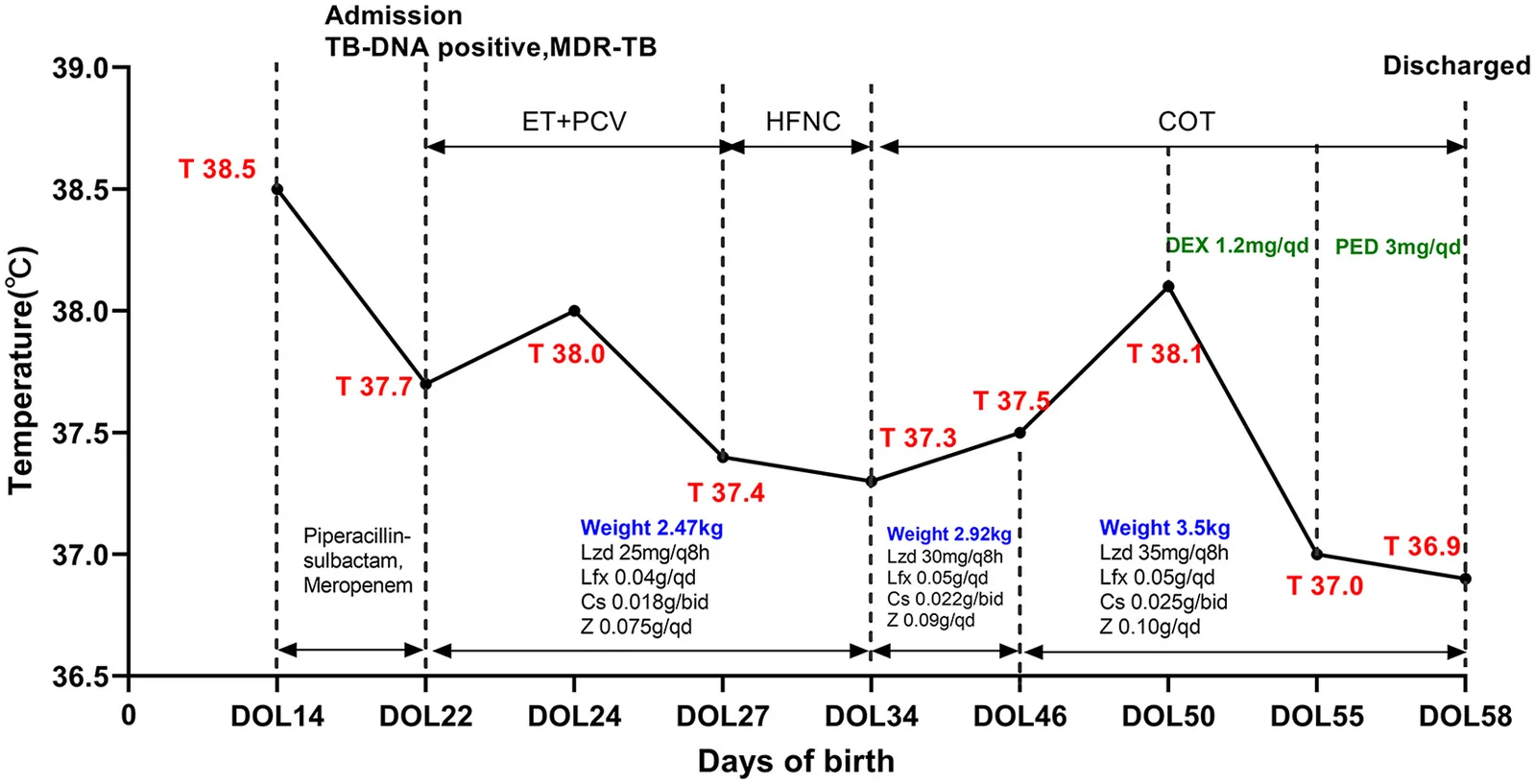
A timeline of treatment and clinical events. Lzd, linazolamide; Lfx, levofloxacin; Cs, cycloserine; Z, pyrazinamide; DEX, dexamethasone; PED, prednisone; ET, endotracheal tube; PCV, pressure-controlled ventilation; SIMV, synchronized intermittent mandatory ventilation; HFNC, high-flow nasal cannula ventilation; COT, conventional oxygen therapy; DOL, day of life.

After admission, she underwent tracheal intubation and ventilation to relieve respiratory distress. A lumbar puncture was performed, and the cerebrospinal fluid (CSF) examination results were the following: white blood cell count 14 × 10^6^/L, total protein 0.56 g/L, glucose 2.99 mmol/L, chlorine 110.5 mmol/L, and adenosine deaminase 0.60 U/L. A TB DNA, acid-fast bacterial smear, and general bacterial culture identification were all negative. GeneXpert Mycobacterium tuberculosis/Rifampicin (MTB/RIF) for gastric juice was positive, and the detection level was “moderate” with rifampicin resistance detected. The acid-fast bacterial smear and TB culture for gastric juice were also positive. The molecular detection of TB DNA in the gastric juice indicated that the strain was resistant to isoniazid, rifampicin, ethambutol, and streptomycin, but susceptible to fluoroquinolones. Her peripheral blood was also sent for metagenomic next-generation sequencing (mNGS), and 130 *Mycobacterium tuberculosis complex* DNA sequences were detected. The chest X-ray showed scattered spots in both lungs (DOL25; Figure 1A), while the chest computed tomography (CT) scan showed diffuse, patchy, high-density shadows with blurred boundaries in both lungs (DOL41; Figure 2A). An ultrasound detected a small amount of pericardial effusion and no obvious abnormalities in the liver or spleen. Disseminated MDR-TB was confirmed. Linezolid (10 mg/kg/q8h), pyrazinamide (30 mg/kg/qd), cycloserine (7.5 mg/kg/bid), and levofloxacin (15 mg/kg/qd) were prescribed for antituberculosis treatment (ATT; DOL22). Her symptoms were relieved. The dosage of anti-TB drugs was adjusted according to her body weight. On the 47th day of life, she had a fever again, which was suspected to be caused by a Herxheimer-like reaction. However, repeated chest X-rays showed absorption of the lesion (DOL47; Figure 1B). A short course of steroids was administered, and her fever disappeared. At DOL58, she was discharged, and her ATT was still ongoing (Figure 3). A subsequent phenotypic drug-susceptible test (DST) showed that the strain was resistant to isoniazid, rifampicin, ethambutol, and streptomycin. A follow-up CT scan 3 months later showed further resorption of the lesion (DOL91; Figure 2B).

**Figure 4:**
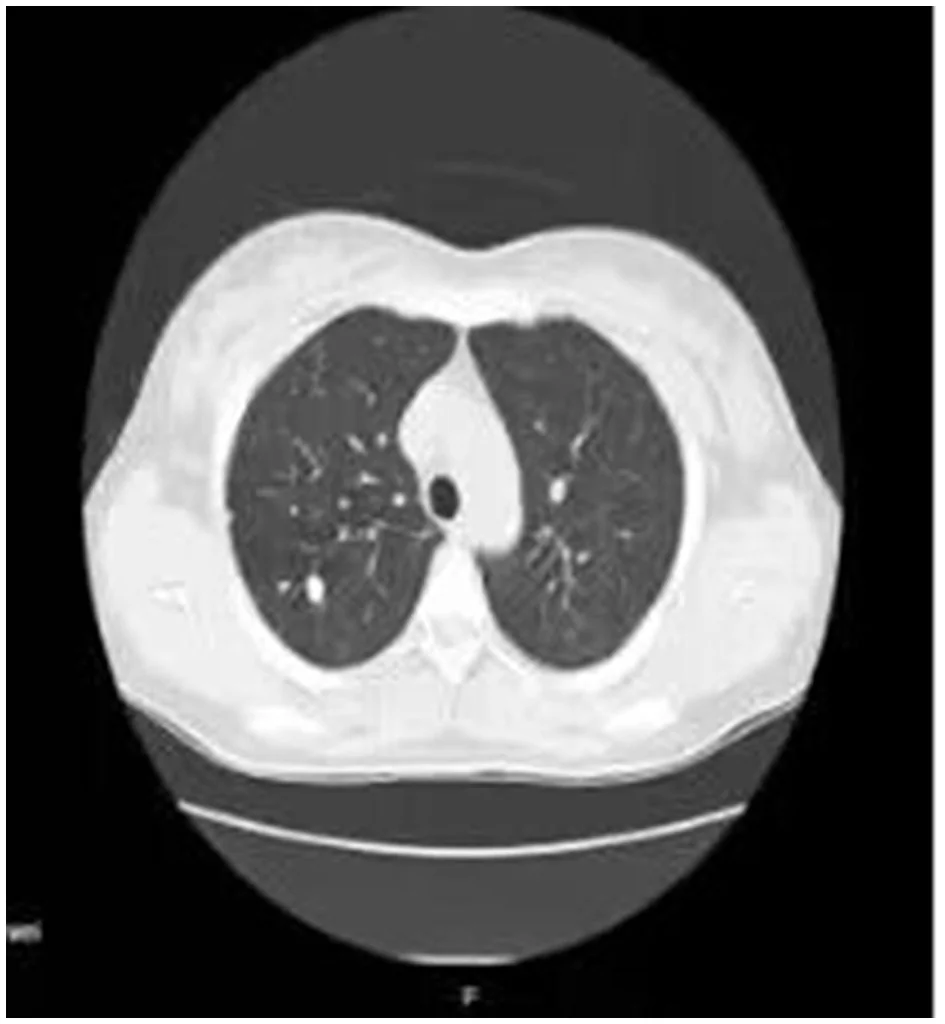
Chest computed tomography of the mother showing multiple nodular shadows.

When the baby was transferred to our hospital and congenital TB was suspected, we found out that the neonate's mother had been infertile for 10 years before undergoing *in vitro* fertilization and embryo transfer (IVF-ET). She had no TB symptoms during pregnancy or even after delivery. However, her chest CT scan showed multiple nodular and striped shadows in the upper lobes (Figure 4). She then underwent a bronchoscopy. Bronchial lavage fluid was sent for mNGS, and 6,288 *Mycobacterium tuberculosis complex* DNA sequences were detected. The TB culture of the mother's vaginal secretions and bronchial lavage fluid were both positive. The DST also found that the strains were resistant to isoniazid, rifampicin, ethambutol, and streptomycin. She was given ATT with bedaquiline, linezolid, levofloxacin, cycloserine, and pyrazinamide. Other family members of the baby also underwent TB screening, and active pulmonary TB and TB infection were ruled out. As of this publication, the baby and her mother's anti-TB treatment are still ongoing.

## Discussion

TB of the female reproductive system makes up approximately 10% of extrapulmonary TB cases and is a leading contributor to infertility. For infertile women, IVF is an effective treatment option, but if infertility is caused by reproductive system TB, receiving IVF-ET may increase the risk of TB dissemination and lead to serious maternal and neonatal morbidity (5). In addition, the premature birth rate of newborns in women with reproductive system tuberculosis is up to 65% (6). Due to premature delivery and immature immune systems and blood–brain barrier function, TB makes newborns prone to multiple organ infections, leading to misdiagnosis and a poor prognosis (7).

Infants with congenital TB typically exhibit symptoms 2–4 weeks after birth, with an average onset age of 24 days (8). The most common clinical symptoms of congenital tuberculosis include anorexia, fever, weight loss, cough, respiratory distress, purulent ear discharge, hepatosplenomegaly, lymphadenopathy, and ascites (1, 8). Due to the hematogenous transmission pattern of congenital tuberculosis and the physiological characteristics of infected newborns, it is more prone to central nervous system (CNS) involvement. CSF examination and CNS images in time could help the evaluation of CNS involvement.

Congenital pulmonary tuberculosis imaging presents non-specific characteristics. Early lesions appearing as interstitial lesions and diffuse miliary shadows might be a clue for diagnosing severe disseminated TB. Widely distributed nodules and patchy shadows are usually considered disease progression (9). The World Health Organization recommends using GeneXpert as the primary screening tool for children suspected of having pulmonary TB and suspected of having MDR pulmonary TB. Additionally, the WHO recommends using Xpert MTB/RIF to detect Mycobacterium tuberculosis in non-respiratory tract samples, such as cerebrospinal fluid and lymph nodes, for the diagnosis of extrapulmonary tuberculosis. For the diagnosis of congenital tuberculosis, pathogenic tests such as body fluid culture, acid-fast staining, and tissue biopsy are considered the gold standard (10). The early diagnosis of MDR-TB in our patient relied on the molecular test, which was consistent with the final phenotypic results.

Congenital TB tends to progress rapidly; therefore, early identification of the pathogen and prompt treatment decrease the mortality associated with the infection (11). At present, the four first-line drugs for TB are isoniazid, rifampicin, pyrazinamide, and ethambutol (12). Although the optimal duration of therapy has not yet been determined, for miliary TB and CNS TB, many experts recommend a treatment duration of 12 months (13). However, for infants with MDR, or extensively drug-resistant TB, the WHO currently recommends a scheduled treatment program similar to that for adults. Moreover, due to the rapid growth and development of newborns, it is recommended to adjust the medication dosage in a timely manner based on their weight every month (14). If adverse reactions occur during the medication process, the treatment plan should be adjusted according to the severity of the adverse reactions (15).

We can summarize some experience from this case: (1) Women who are infertile should be screened for TB before undergoing IVF-ET. If diagnosed with reproductive system TB, they should receive standard ATT to decrease the risk of TB dissemination infection. (2) Children with congenital TB tend to be born prematurely, have a low birth weight, and usually have symptom onset at 2–4 weeks after birth. (3) Newborns who have an unexplained fever or worsening respiratory symptoms should consider the possibility of atypical pathogens like TB. (4) Congenital TB in children often involves multiple organs, such as the lungs, liver, digestive tract, brain, blood, and so on. It is necessary to screen for infection throughout the whole body, particularly for a possible CNS infection. Body fluids such as gastric juice, sputum, and cerebrospinal fluid for smear, culture, and molecular tests can confirm the diagnosis if any test is positive. (5) For MDR-TB, the regimen should be used based on the results of drug susceptibility tests to ensure at least four effective drugs are included.

In summary, congenital MDR-TB is extremely rare and difficult to distinguish from other infections in early diagnosis. Maternal history and maternal exposure will facilitate early diagnosis, especially in epidemic areas. Early recognition and early treatment can prevent serious consequences.

## Data availability statement

The original contributions presented in the study are included in the article/Supplementary material, further inquiries can be directed to the corresponding author.

## Ethics statement

Ethical approval was not required for the study involving humans in accordance with the local legislation and institutional requirements. Written informed consent to participate in this study was not required from the participants or the participants' legal guardians/next of kin in accordance with the national legislation and the institutional requirements. Written informed consent was obtained from the individual(s), and minor(s)' legal guardian/next of kin, for the publication of any potentially identifiable images or data included in this article.

## Author contributions

HZ: Writing – original draft. LX: Writing – review & editing. PZ: Supervision, Writing – review & editing.
